# Additive effect of evaluating microsurface and microvascular patterns using magnifying endoscopy with narrow-band imaging in gastric cancer: a post-hoc analysis of a single-center observational study

**DOI:** 10.1186/s12876-022-02197-x

**Published:** 2022-03-16

**Authors:** Yusuke Horiuchi, Toshiaki Hirasawa, Naoki Ishizuka, Junki Tokura, Mitsuaki Ishioka, Yoshitaka Tokai, Ken Namikawa, Shoichi Yoshimizu, Akiyoshi Ishiyama, Toshiyuki Yoshio, Junko Fujisaki

**Affiliations:** 1 Department of Gastroenterology, Cancer Institute Hospital of Japanese Foundation for Cancer Research, 3-8-31 Ariake, Koto-ku, Tokyo, 135-8550 Japan; 2 Department of Clinical Trial Planning and Management, Cancer Institute Hospital of Japanese Foundation for Cancer Research, Tokyo, Japan

**Keywords:** Diagnostic performance, Endoscopic submucosal dissection, Endoscopy, Gastric cancer

## Abstract

**Background:**

No studies have compared the performance of microvascular and microsurface patterns alone with their combination in patients undergoing magnifying endoscopy with narrow-band imaging for diagnosing gastric cancer. This study aimed to clarify the differences in diagnostic performance among these methods.

**Methods:**

Thirty-three participating endoscopists who had received specialized training in magnifying endoscopy evaluated the microvascular and microsurface patterns of images of 106 cancerous and 106 non-cancerous lesions. If classified as “irregular,” the lesion was diagnosed as gastric cancer. To evaluate diagnostic performance, we compared the diagnostic accuracy, sensitivity, and specificity of these methods.

**Results:**

Performance-related items did not differ significantly between microvascular and microsurface patterns. However, the diagnostic accuracy and sensitivity were significantly higher when using a combination of these methods than when using microvascular (82.1% [76.4–86.7] *vs*. 76.4% [70.3–81.6] and 69.8% [60.5–77.8] *vs*. 63.2% [53.7–71.8]; *P* < 0.001 and *P* = 0.008, respectively) or microsurface (82.1% [76.4–86.7] *vs*. 73.6% [67.3–79.1] and 69.8% [60.5–77.8] *vs*. 52.8% [43.4‒62.1]; both, *P* < 0.001) patterns alone. The additive effect on diagnostic accuracy and sensitivity was 5.7‒8.6% and 6.6‒17.0%, respectively.

**Conclusions:**

We demonstrate the superiority of the combination of microvascular and microsurface patterns over microvascular or microsurface patterns alone for diagnosing gastric cancer. Our data support the use of the former method in clinical practice. Although a major limitation of this study was its retrospective, single-center design, our findings may help to improve the diagnosis of gastric cancer.

## Background

Gastric cancer is among the most prevalent types of cancer and is associated with high mortality [1, 2]. However, owing to recent advances in endoscopic technology, the number of gastric cancer cases detected at an early stage has increased and mortality rates have decreased [3–5]. Among these recent developments is magnifying endoscopy with narrow-band imaging (ME-NBI), which has superior diagnostic performance when combined with conventional endoscopy [6, 7]. Therefore, ME-NBI is currently the standard method for diagnosing gastric cancer.

Previous studies have reported that the vessel plus surface (VS) classification system used during ME-NBI is useful for diagnosing gastric cancer [7, 8]. In the VS classification system [7, 8], the demarcation between the cancerous and non-cancerous tissues is identified under low magnification, following which the target area is observed under high magnification, and both the microvascular (MV) and microsurface (MS) patterns are evaluated. Cancer is diagnosed if irregular findings are observed with respect to either the MV or MS pattern. Meanwhile, several studies have reported that assessments based on MV patterns alone are feasible for diagnosing gastric cancer [9–11]. To date, no study has compared the diagnostic performance of the combination of MS and MV with MV alone; therefore, this difference is unclear. In addition, as for MS alone, there are no reports of its usefulness.

If there is indeed an additive effect of evaluating MS and MV, the results of this analysis can demonstrate the superiority of combining MS and MV over MV alone or MS alone for diagnosing gastric cancer. In contrast, if high diagnostic performance can be achieved with MS or MV alone, the diagnostic system can be simplified by omitting the unnecessary component. In this study, we evaluated the respective contributions of MS and MV patterns in diagnosing gastric cancer and assessed the differences in diagnostic performance between the combination of MS and MV and MS or MV alone.

## Methods

This study was a post-hoc analysis of a single-center observational study [12]. We used images of 118 consecutive lesions in 114 patients who underwent endoscopic submucosal dissection (ESD) performed by a single endoscopist (Y.H.) between July 2016 and July 2019. Patient images and data were extracted from electronic medical records. The study design was approved by the Institutional Review Board of the Cancer Institute Hospital of Japanese Foundation for Cancer Research, Tokyo, Japan (approval number: 2019-1032). Informed consent was obtained from each patient for the use of pathological specimens and imaging data.

ME-NBI was performed before treatment (on a different day). Before examination, a soft hood (MB-46; Olympus Medical Systems, Tokyo, Japan) was mounted on the tip of the endoscope to enable the endoscopist to consistently fix the mucosa at approximately 2 mm. We first performed endoscopy with white-light imaging, following which ME-NBI was performed to identify cancerous and noncancerous tissues. In particular, the demarcation between the cancerous and noncancerous tissues was identified under low magnification, following which the target area was observed under high magnification. Finally, endoscopy was performed following indigo carmine spraying.

The inclusion criterion was the availability of ME-NBI images at the utmost oral side of the cancerous tissue and adjacent noncancerous tissue (one image each of the cancerous and noncancerous tissue per patient). The exclusion criteria included a lack of ME-NBI images and unclear images owing to the presence of mucus, blood, or halation.

In accordance with the Gastric Cancer Treatment Guidelines [13], we compared the pathological macro images that revealed the cancerous areas from the pathological reports to the endoscopic images, confirming the cancerous and noncancerous areas in all cases based on post-ESD pathological findings, which are considered the gold standard. The diagnosis of gastric cancer was assessed according to the Japanese classification of gastric carcinoma [14]. All images were selected by a single instructor (YH) of the Japan Gastroenterological Endoscopy Society. Another instructor of the Japan Gastroenterological Endoscopy Society (T.H.) confirmed that all images met the inclusion criteria. GIF-H260Z and GIF-H290Z systems (Olympus Medical Systems, Tokyo, Japan) were used for ME-NBI.

### Endoscopists involved in diagnostic imaging and the diagnostic method

Thirty-three endoscopists with specialized training in ME-NBI across 19 institutions participated in the diagnostic process. The VS classification system was used for the diagnosis of lesions [7, 8]. The VS classification system was established based on diagnoses made by endoscopists with ME-NBI training at specialized facilities [7]. It was previously reported that diagnostic performance is better among such endoscopists than among those without training [15]. Since diagnoses made by endoscopists without specialized training in ME-NBI may not adequately reflect the accuracy of ME-NBI diagnosis, endoscopists with specialized training in ME-NBI were selected.

**Fig. 1:**
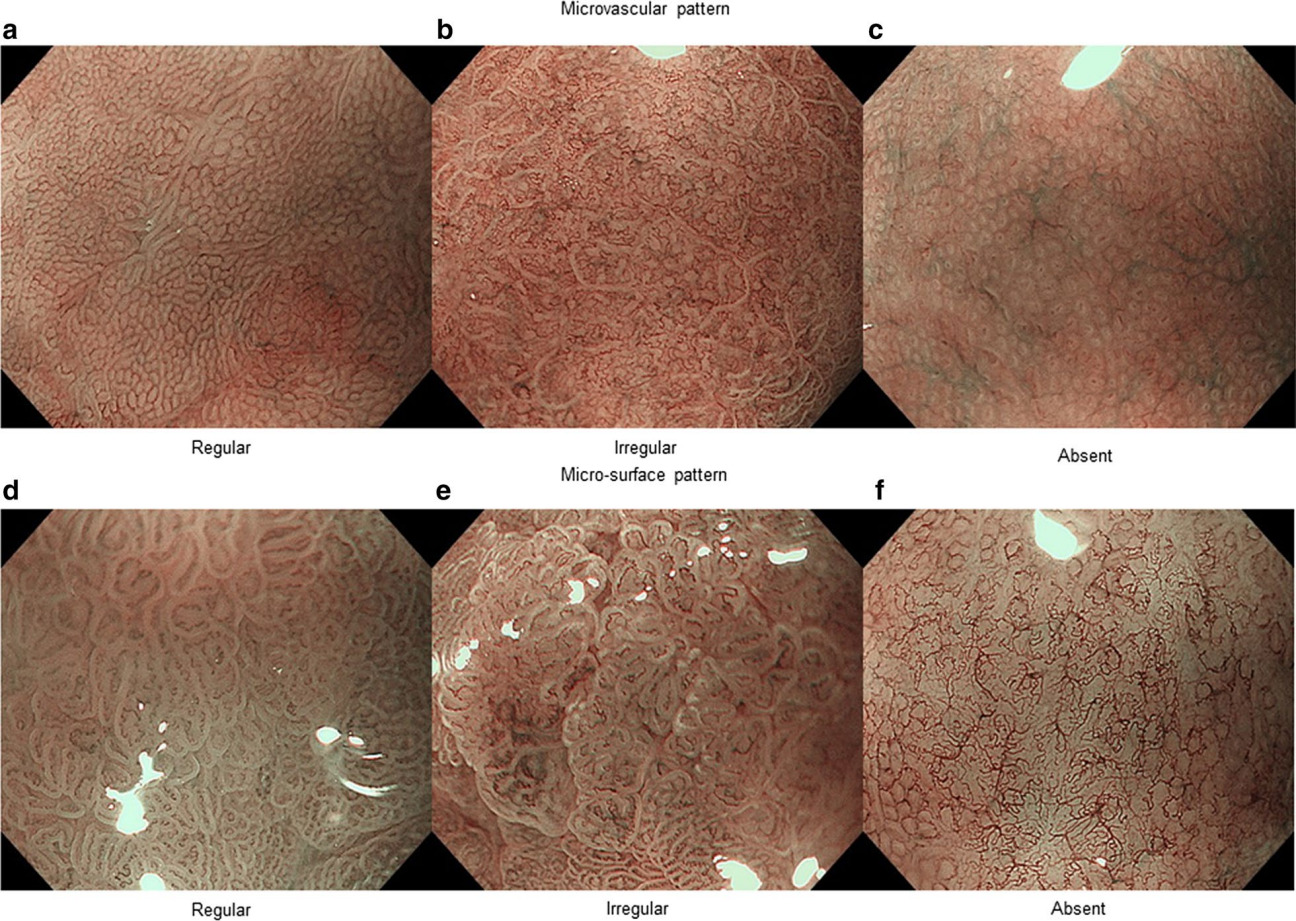
Microvascular pattern and microsurface pattern classification. The vessel plus surface classification system is used to distinguish between cancerous and non-cancerous tissues in patients undergoing magnifying endoscopy with narrow-band imaging. If either the microvascular pattern (V) or the microsurface pattern (S) is classified as “irregular,” the lesion is diagnosed as cancerous. The details of the classification system are as follows: microvascular pattern: **a** uniform blood vessels (“regular”); **b** blood vessels that expand locally and are non-uniform (“irregular”); and **c** no vascular findings (“absent”). microsurface pattern: **d** uniform surface structure (“regular”); **e** non-uniform surface structure of different sizes (“irregular”); and **f** no surface structure (“absent”)

Each endoscopist evaluated each image using the terms “regular,” “irregular,” “absent,” or “inconclusive.” If either MV or MS was defined as “irregular,” the lesion was diagnosed as cancerous; the lesion was not diagnosed as cancerous for any of the other definitions. Representative ME-NBI images in which the VS classification system was used are shown in Fig. 1. MV was defined as “irregular” if the shape (such as closed-loop [polygonal], open loop, tortuous, branched, bizarrely shaped, and network) and size of the vessels varied and their arrangement and distribution were irregular [7]. MS was defined as “irregular” if the individual morphology of the crypt epithelium exhibited irregular tubular/linear/curved/papillary/villous structures, varying in width and length, and their arrangement and distribution were irregular [7].

In our previous study [12], the diagnosis of cancer or non-cancer provided by each endoscopist was aggregated to calculate the diagnostic performance among all participating endoscopists. In this study, the diagnostic results (“regular,” “irregular,” “absent,” or “inconclusive”) for MS and/or MV were collected for each image using the original data from our previous study [12], and the diagnostic performance based on MS and/or MV was calculated for all images.

### Evaluation criteria

This study was conducted in accordance with the Standards for the Reporting of Diagnostic Accuracy Studies 2015 guidelines [16]. We recently examined images combining endocytoscopy with NBI in another post-hoc analysis of data collected in the single-center observational study noted above [12], and the same methods of analysis were used in this study [17]. Briefly, for each image, the classification (“regular,” “irregular,” “absent,” or “inconclusive”) with the greatest frequency of response among the endoscopists was regarded as the final diagnosis (we did not set a threshold). When multiple classifications exhibited the maximum number of responses, the final diagnosis was regarded as “inconclusive.” In contrast, if the agreement rate among the endoscopists for each image was low, the reliability of the diagnosis was considered low, and generalization was difficult. Therefore, we also calculated the diagnostic agreement rate for each image, which was defined as the ratio of the maximum number of responses for a given classification to the total number of responses.

We calculated the median and interquartile range (IQR) of the diagnostic agreement rate for all images, which was confirmed based on the MV and MS. MV and MS diagnoses were then aggregated for each of the cancerous and non-cancerous images.

Based on the aggregated results, we calculated the diagnostic accuracy, sensitivity, specificity, positive predictive value (PPV), and negative predictive value (NPV) for the diagnosis based on the MV alone (irregular pattern indicative of cancer), the MS alone (irregular pattern indicative of cancer), and the combination of MS and MV (cancer diagnosed if either pattern was considered irregular). Subsequently, we compared the diagnostic performance between MV alone and MS alone, between MV alone and the combination of MS and MV, and between MS alone and the combination of MS and MV. The classification of “inconclusive” was considered an incorrect diagnosis.

In addition, to identify factors contributing to the additive effect of imaging patterns, we compared cancerous lesion characteristics (location, macroscopic type, tumor diameter, depth, ulcerative findings, histological type, and a history of *Helicobacter pylori* [*H. pylori*] infection) between patients correctly diagnosed using MV alone and those correctly diagnosed using MS alone. The cutoff values for tumor diameter were determined with reference to the median value for all tumors.

*H. pylori*-uninfected cases were defined as meeting all of the following criteria: (1) no prior *H. pylori* eradication, (2) negative urea breath test results (UBIT; Otsuka, Tokushima, Japan), (3) negative results for *H. pylori* antibodies (*H. pylori* antibody II: EIKEN Co. Ltd., Tokyo, Japan), (4) negative pepsinogen (PG) test results (positive cutoff value: PGI ≤ 70 ng/mL; PGI/II ratio ≤ 3), (5) endoscopically confirmed positive regular arrangement of collecting venules in the lower gastric body [18], and (6) histologically confirmed *H. pylori*-uninfected case and negative inflammatory cell infiltration result based on the updated Sydney system [19]. Patients that did not meet these criteria were considered to have a history of *H. pylori* infection.

The inclusion criteria for the *H. pylori*-infected with eradication group were as follows: negative for *H. pylori* antibodies or a negative C urea breath test result, if the patient underwent *H. pylori* eradication at our hospital or another hospital, with a confirmed negative urea breath test result ≥ 4 weeks after initiating *H. pylori* eradication, if the patient was positive for *H. pylori* antibodies or had a positive urea breath test result at the first examination at our hospital. The *H. pylori*-infected without eradication group did not meet these criteria and was labeled the “noneradication group.”

### Statistical analysis

The median and IQR were used when calculating the diagnostic agreement rate for all images. McNemar tests with 95% confidence intervals (CIs) were used to compare the diagnostic accuracy, sensitivity, and specificity among diagnoses made based on MV alone, MS alone, and the combination of MS and MV. Fisher’s exact tests with 95% CIs were used to compare the PPV and NPV among the three diagnostic methods. Fleiss’ kappa statistic was used to assess the diagnostic agreement between images.

Statistical significance was set at *P* < 0.05/3 using the Bonferroni correction for pairwise comparisons (MV alone *vs*. MS alone, MV alone *vs*. the combination of MS and MV, and MS alone *vs*. the combination of MS and MV). Fisher’s exact test was used to compare lesion characteristics between patients correctly diagnosed based on MV alone and those correctly diagnosed based on the combination of MS and MV, with statistical significance set at *P* < 0.05. JMP version 13.2 (SAS Institute, Cary, NC, USA) was used to perform the analyses.

## Results

As in our previous study [12], a total of 106 lesions in 102 patients satisfied the inclusion and exclusion criteria. The patient characteristics are summarized in Table 1. The median age was 71 (IQR, 61.8–77.0; range, 26–87) years; 66 (64.7%) patients were male and 36 (35.3%) were female. Endoscopic images of the adjacent non-cancerous tissue revealed that eight lesions (7.5%) exhibited atrophy, intestinal metaplasia, and erosion; 44 (41.5%) exhibited atrophy and intestinal metaplasia; two (1.9%) exhibited atrophy and erosion; 40 (37.7%) exhibited atrophy alone; and 12 (11.3%) exhibited none of the above.

**Table 1:** Patient characteristics

	102 cases, 106 lesions
Age (years)	71 (61.8–77.0) [26–87]
Sex (male/female)	66 (64.7)/36 (35.3)
Location	
Upper third	26 (24.5)
Middle third	59 (55.7)
Lower third	17 (16.0)
Gastric tube^a^	4 (3.8)
Macroscopic type	
Elevated	15 (14.2)
Flat	5 (4.7)
Depressed	83 (78.3)
Complex	3 (2.8)
Tumor diameter (mm)	14 (9.0–20.3) (1.5–57.0)
Depth	
Intramucosal	90 (84.9)
Submucosal	
< 500 μm	12 (11.3)
≥ 500 μm	4 (3.8)
Ulcerative findings	
Present	5 (4.7)
Absent	101 (95.3)
Histological type	
Differentiated	82 (77.4)
Undifferentiated	24 (22.6)
History of *Helicobacter pylori* infection	
Infected	97 (91.5)
Noneradicated	23 (21.7)
Eradiated	74 (76.3)
Uninfected	9 (8.5)
Endoscopic findings in adjacent noncancerous tissue	
Atrophy	94 (88.7)
Intestinal metaplasia	52 (49.1)
Erosion	10 (9.4)
None of the above	12 (11.3)

Data are presented as numbers (%), except for tumor size, which is expressed as median (interquartile range) [range]

^a^A gastric tube is a reconstruction method used after esophagectomy

The Patients had undergone ME-NBI before ESD on a different day. Thirty-three endoscopists with specialized training analyzed and provided a diagnosis (“regular,” “irregular,” “absent,” or “inconclusive”) based on the MS and/or MV pattern of each image. Table 2 shows the diagnostic agreement rate for all images (72.7% for MV [IQR: 60.6–81.8]; 72.7% for MS [IQR: 54.5–81.8]). The lower limit of the IQR for both MV and MS was > 50%. The Fleiss’ kappa statistic for all images was approximately 0.3 for both MV and MS.

**Table 2:** Median diagnostic agreement rate for each finding in all images

	Diagnostic agreement rate	kappa statistic
MV		
Total (n = 212)	72.7% (60.6–81.8%) [36.4–100.0%]	0.320
Cancerous tissue (n = 106)	72.7% (57.6–87.9%) [36.4–100.0%]	0.291
Non-cancerous tissue (n = 106)	72.7% (60.6–81.8%) [36.4–93.9%]	0.102
MS		
Total (n = 212)	72.7% (54.5–81.8%) [30.3–93.9%]	0.289
Cancerous tissue (n = 106)	60.6% (51.5–78.8%) [33.3–93.9%]	0.261
Non-cancerous tissue (n = 106)	78.8% (63.6–84.8%) [30.3–93.9%]	0.092

Data are expressed as median (interquartile range) [range]

MS, microsurface pattern; MV, microvascular pattern

Table 3 shows the diagnoses made using a combination of MS and MV in cancerous and non-cancerous lesion images. Among the noncancerous lesion images, regular MS and MV findings were observed in 94 patients (88.7%). Among the images of cancerous cases, irregular MV and MS findings were observed in 49 images (46.2%), irregular MV findings were observed only in 18 images (16.9%), and irregular MS findings were observed only in seven images (6.6%).

**Table 3:** Combination of MS and MV diagnoses in images of cancerous and non-cancerous lesions

MV	MS	Non-cancerous (n = 106)	Cancerous (n = 106)
Regular	Regular	94 (88.7)	29 (27.4)
	Irregular	0	5 (4.7)
	Absent	0	1 (0.9)
	Inconclusive	0	0
Irregular	Regular	0	1 (0.9)
	Irregular	6 (5.7)	49 (46.2)
	Absent	0	17 (16.0)
	Inconclusive	0	0
Absent	Regular	1 (0.9)	1 (0.9)
	Irregular	0	0
	Absent	0	0
	Inconclusive	0	0
Inconclusive	Regular	5 (4.7)	0
	Irregular	0	2 (1.9)
	Absent	0	0
	Inconclusive	0	1 (0.9)

Data are presented as numbers (%)

MS, microsurface pattern; MV, microvascular pattern

Based on the data presented in Table 3, we calculated the diagnostic accuracy, sensitivity, specificity, PPV, and NPV. Subsequently, we compared the diagnostic performance between MV alone and MS alone, between MV alone and the combination of MS and MV, and between MS alone and the combination of MS and MV (Table 4). There was no significant difference in the diagnostic performance of any of the items between MV alone and MS alone. In contrast, diagnostic accuracy and sensitivity were significantly higher for the combination of MS and MV than that for MV alone and MS alone (diagnostic accuracy: 82.1% [95% CI: 76.4–86.7] *vs*. 76.4% [95% CI: 70.3–81.6] and 82.1% [95% CI: 76.4–86.7] *vs*. 73.6% [67.3–79.1], both *P* < 0.001; and sensitivity: 69.8% [95% CI: 60.5–77.8] *vs*. 63.2% [95% CI: 53.7–71.8] and 69.8% [95% CI: 60.5–77.8] *vs*. 52.8% [43.4‒62.1], *P* = 0.008 and *P* < 0.001, respectively). The additive effect on diagnostic accuracy and sensitivity was 5.7‒8.6% and 6.6‒17.0%, respectively.

**Table 4:** Comparison of diagnostic performance

	1. MV alone	2. MS alone	3. MV and MS	*P* (1 *vs*. 2)	*P* (1 *vs*. 3)	*P* (2 *vs*. 3)
Accuracy, % (95% CI)	76.4 (70.3–81.6)	73.6 (67.3–79.1)	82.1 (76.4–86.7)	0.027	< 0.001	< 0.001
Sensitivity, % (95% CI)	63.2 (53.7–71.8)	52.8 (43.4–62.1)	69.8 (60.5–77.8)	0.028	0.008	< 0.001
Specificity, % (95% CI)	89.6 (82.4–94.1)	94.3 (88.2–97.4)	94.3 (88.2–97.4)	0.025	0.025	> 0.99
PPV, % (95% CI)	91.8 (83.2–96.2)	90.2 (80.2–95.4)	92.4 (84.4–96.5)	0.770	> 0.99	0.763
NPV, % (95% CI)	71.9 (64.0–78.7)	66.2 (58.4–73.3)	75.2 (67.2–81.8)	0.312	0.584	0.118

CI, confidence interval; MV, microvascular pattern; MS, microsurface pattern; NPV, negative predictive value; PPV, positive predictive value

In addition, as shown in Table 5, there were no significant differences in cancerous lesion characteristics between lesions correctly diagnosed using MV alone and those correctly diagnosed using MS alone.

**Table 5:** Comparison of cancerous characteristics between patients correctly and incorrectly diagnosed using MV alone

	Correctly diagnosed by MV alone (n = 67)	Incorrectly diagnosed by MV alone (requiring MS) (n = 7)	*P* value
Location			
Upper third	7 (10.5)	2 (28.6)	0.4759
Middle third	40 (59.7)	3 (42.9)	
Lower third	16 (23.9)	2 (28.6)	
Gastric tube	4 (6.0)	0	
Macroscopic type			
Elevated	11 (16.4)	1 (14.3)	0.7951
Flat	5 (7.5)	0	
Depressed	48 (71.6)	6 (85.7)	
Complex	3 (4.5)	0	
Tumor diameter			
≥ 15 mm	33 (49.3)	1 (14.3)	0.1158
< 15 mm	34 (50.7)	6 (85.7)	
Depth			
Intramucosal	57 (85.1)	6 (85.7)	> 0.9999
Submucosal invasion	10 (14.9)	1 (14.3)	
Ulcerative findings			
Present	3 (4.5)	2 (28.6)	0.0674
Absent	64 (95.5)	5 (71.4)	
Histological type			
Differentiated	50 (74.6)	6 (85.7)	> 0.9999
Undifferentiated	17 (25.4)	1 (14.3)	
History of *Helicobacter pylori* infection			
Infected			0.2389
Non-eradicated	15 (22.4)	0	
Eradicated	47 (70.2)	7 (100)	
Uninfected	5 (7.5)	0	

Data are presented as numbers (%)

MS, microsurface pattern; MV, microvascular pattern

## Discussion

To our knowledge, this study is the first to evaluate the respective contributions of MS and MV in diagnosing gastric cancer, as well as the differences in diagnostic performance between the combination of MS and MV and each of these alone.

Our findings indicate that there are cases that cannot be diagnosed without considering MS. Moreover, we observed no significant difference in the diagnostic performance between MV alone and MS alone. In other words, diagnoses determined based on MS may exhibit the same diagnostic performance as those determined based on MV. We are not aware of any previous investigations into the usefulness of MS alone. In addition, the diagnostic accuracy and sensitivity of the combination of MS and MV were significantly higher than those of either MV or MS alone, and an additive effect of MS and MV was observed. The ability to identify cancerous lesions during endoscopic screening is paramount. Therefore, sensitivity is the most important factor contributing to diagnostic performance. The major strength of our study is that our findings highlight the importance of MS when diagnosing gastric cancer in clinical practice.

While the combination of MS and MV is reportedly useful for diagnosing gastric cancer [7, 8], some studies have reported that MV alone is feasible for diagnosis [9–11]. Furthermore, no previous reports have compared the diagnostic performance between the combination of MS and MV and MV alone. Given that our results show the superiority of the combination of MS and MV over using MV alone for diagnosing gastric cancer, our data support the application of the former in clinical practice.

Moreover, the lower limit of the IQR for the diagnostic agreement rate for both MV and MS was > 50%. Despite the large number of images (n = 212) and endoscopists (n = 33), the Fleiss’ kappa statistic showed fair agreement for the diagnosis of gastric cancer between MV and MS in all images. The aggregated responses in this study were considered the general responses of choice for the majority of endoscopists. This supports the reliability of the calculated diagnostic performance and the generalizability of our results and is one of the strengths of this study.

We also compared the cancerous lesion characteristics between patients correctly diagnosed using MV alone and those correctly diagnosed using MS to clarify whether the additive effect is associated with specific image attributes. However, no significant differences in any items were observed, which may have been because of the small number of lesions correctly diagnosed based on the combination of MS and MV. While further studies are needed, this result suggests that a diagnosis of gastric cancer should be made based on the combination of both patterns, regardless of lesion characteristics.

This study had some limitations. First, the images were retrospectively collected from a single center. Second, diagnoses were not made at the time of real-time endoscopy. Finally, as all patients underwent ESD, some lesions could have been overlooked. Meanwhile, the images used in this study were from consecutive cases, wherein patients were examined at a specialized cancer hospital, and all images were evaluated by 33 endoscopists from 19 facilities nationwide. Therefore, the influence of bias was likely small, and the results of this study can be generalized. Furthermore, because the design of this study included a distinguishing feature between the cancerous and adjacent non-cancerous tissue, our findings indicated that it is possible to distinguish between adjacent non-cancerous gastric mucosa and gastric cancer, suggesting that our findings may be useful for diagnosing gastric cancer. Therefore, despite the limitations of this study, we consider our results to be clinically meaningful. To address the study limitations, we plan to prospectively evaluate the diagnostic performance (including an evaluation of the demarcating line) of MS alone, MV alone, and the combination of MS and MV during real-time endoscopy in collaboration with multiple centers. The results of this study will provide a valuable basis for comparison.

## Conclusions

Our findings demonstrated that the combination of MS and MV has superior diagnostic accuracy and sensitivity for diagnosing gastric cancer compared to MS or MV alone. Our results support the application of the combination of MS and MV in clinical practice and may contribute to improving the diagnosis of gastric cancer.

## Data Availability

The datasets generated and/or analyzed during the current study are not publicly available due to privacy concerns but are available from the corresponding author on reasonable request.

## Abbreviations

ME-NBI: Magnifying endoscopy with narrow-band imaging
VS: Vessel plus surface
MV: Microvascular pattern
MS: Microsurface pattern
ESD: Endoscopic submucosal dissection
JGES: Japan Gastroenterological Endoscopy Society
IQR: Interquartile range
PPV: Positive predictive value
NPV: Negative predictive value
PG: Pepsinogen
CI: Confidence interval
